# Sickle Cell Disease Treatment with Arginine Therapy (STArT): study protocol for a phase 3 randomized controlled trial

**DOI:** 10.1186/s13063-023-07538-z

**Published:** 2023-08-17

**Authors:** Chris A. Rees, David C. Brousseau, Daniel M. Cohen, Anthony Villella, Carlton Dampier, Kathleen Brown, Andrew Campbell, Corrie E. Chumpitazi, Gladstone Airewele, Todd Chang, Christopher Denton, Angela Ellison, Alexis Thompson, Fahd Ahmad, Nitya Bakshi, Keli D. Coleman, Sara Leibovich, Deborah Leake, Dunia Hatabah, Hagar Wilkinson, Michelle Robinson, T. Charles Casper, Elliott Vichinsky, Claudia R. Morris, the SCD Arginine Study Group and PECARN

**Affiliations:** 1 Department of Pediatrics, Division of Pediatric Emergency Medicine, Emory University School of Medicine, 1760 Haygood Drive NE, Atlanta, GA W45830322 USA; 2 Children’s Healthcare of Atlanta, Atlanta, GA USA; 3 Department of Pediatrics, Nemours Children’s Health Delaware and the Sidney Kimmel Medical College, Thomas Jefferson University, Wilmington, DE USA; 4 Nationwide Children’s Hospital, Columbus, OH USA; 5 Children’s National Hospital, The George Washington University School of Medicine and Health Sciences, Washington, DC USA; 6 Texas Children’s Hospital and Baylor College of Medicine, Houston, TX USA; 7 Children’s Hospital Los Angeles and Keck School of Medicine of the University of Southern California, Los Angeles, CA USA; 8 Children’s Hospital of Philadelphia, Philadelphia, PA USA; 9 Washington University in St. Louis, St. Louis, MO USA; 10 Division of Pediatric Hematology/Oncology/BMT, Department of Pediatrics, Emory University School of Medicine, Atlanta, GA USA; 11 Aflac Cancer and Blood Disorders Center, Children’s Healthcare of Atlanta, Atlanta, GA USA; 12 Medical College of Wisconsin and Children’s Wisconsin, Milwaukee, WI USA; 13 University of California, San Francisco, CA USA; 14 University of Utah School of Medicine, Salt Lake City, UT USA; 15 Center for Maternal-Fetal Precision Medicine, University of California, San Francisco, CA USA; 16 Department of Pediatrics, UCSF-Benioff Children’s Hospital-Oakland, Oakland, CA USA

**Keywords:** Sickle cell disease, Arginine, Vaso-occlusive pain, Randomized controlled trial

## Abstract

**Background:**

Despite substantial illness burden and healthcare utilization conferred by pain from vaso-occlusive episodes (VOE) in children with sickle cell disease (SCD), disease-modifying therapies to effectively treat SCD-VOE are lacking. The aim of the Sickle Cell Disease Treatment with Arginine Therapy (STArT) Trial is to provide definitive evidence regarding the efficacy of intravenous arginine as a treatment for acute SCD-VOE among children, adolescents, and young adults.

**Methods:**

STArT is a double-blind, placebo-controlled, randomized, phase 3, multicenter trial of intravenous arginine therapy in 360 children, adolescents, and young adults who present with SCD-VOE. The STArT Trial is being conducted at 10 sites in the USA through the Pediatric Emergency Care Applied Research Network (PECARN). Enrollment began in 2021 and will continue for 5 years. Within 12 h of receiving their first dose of intravenous opioids, enrolled participants are randomized 1:1 to receive either (1) a one-time loading dose of L-arginine (200 mg/kg with a maximum of 20 g) administered intravenously followed by a standard dose of 100 mg/kg (maximum 10 g) three times a day or (2) a one-time placebo loading dose of normal saline followed by normal saline three times per day at equivalent volumes and duration as the study drug. Participants, research staff, and investigators are blinded to the participant’s randomization. All clinical care is provided in accordance with the institution-specific standard of care for SCD-VOE based on the 2014 National Heart, Lung, and Blood Institute guidelines. The primary outcome is time to SCD-VOE pain crisis resolution, defined as the time (in hours) from study drug delivery to the last dose of parenteral opioid delivery. Secondary outcomes include total parental opioid use and patient-reported outcomes. In addition, the trial will characterize alterations in the arginine metabolome and mitochondrial function in children with SCD-VOE.

**Discussion:**

Building on the foundation of established relationships between emergency medicine providers and hematologists in a multicenter research network to ensure adequate participant accrual, the STArT Trial will provide definitive information about the efficacy of intravenous arginine for the treatment of SCD-VOE for children.

**Trial registration:**

The STArT Trial was registered in ClinicalTrials.gov on April 9, 2021, and enrollment began on June 21, 2021 (NCT04839354).

**Supplementary Information:**

The online version contains supplementary material available at 10.1186/s13063-023-07538-z.

## Administrative information

**Table:** 

Title {1}	Sickle Cell Disease Treatment with Arginine Therapy (STArT): Study Protocol for a Phase 3 Randomized Controlled Trial
Trial registration {2a and 2b}	The STArT Trial was registered in ClinicalTrials.gov on April 9, 2021, and enrollment began on June 21, 2021 (NCT04839354)
Protocol version {3}	v1.03 dated 1.18.2023
Funding {4}	This study was supported by the NIH/NHLBI under Award Number 5UH3HL148560 (to CRM), and in part by NIH/NCCIH K24AT009893 (to CRM) and the Pediatric Emergency Care Applied Research Network (PECARN), supported by the Health Resources and Services Administration (HRSA) of the U.S. Department of Health and Human Services (HHS), in the Maternal and Child Health Bureau (MCHB), under the Emergency Medical Services for Children (EMSC) program through the following cooperative agreements: DCC-University of Utah, GLEMSCRN-Nationwide Children’s Hospital, HOMERUN-Cincinnati Children’s Hospital Medical Center, PEMNEWS-Columbia University Medical Center, PRIME-University of California at Davis Medical Center, CHaMP node-State University of New York at Buffalo, WPEMR-Seattle Children's Hospital, and SPARC-Rhode Island Hospital/Hasbro Children's Hospital. NB received funding from the NIH/NHLBI under award number 1K23HL140142 and 1K23HL140142-03S1, from the Doris Duke Charitable Foundation COVID19 Fund to Retain Clinical Scientists-PeRSEVERE Program at Emory University School of Medicine, and the Georgia Clinical and Translational Science Alliance under award UL1-TR002378
Author details {5a}	Chris A. Rees,^1,2^ David C. Brousseau,^3^ Daniel M. Cohen,^4^ Anthony Villella,^4^ Carlton Dampier,^1,2^ Kathleen Brown,^5^ Andrew Campbell,^5^ Corrie E. Chumpitazi,^6^ Gladstone Airewele,^6^ Todd Chang,^7^ Christopher Denton,^7^ Angela Ellison,^8^ Alexis Thompson,^8^ Fahd Ahmad,^9^ Nitya Bakshi,^10,11^ Keli D. Coleman,^12^ Sara Leibovich,^13^ Deborah Leake,^2^ Dunia Hatabah,^1^ Hagar Wilkinson,^2^ Michelle Robinson,^14^ T. Charles Casper,^14^ Elliott Vichinsky,^15,16^ Claudia R. Morris^1,2^: on behalf of the SCD Arginine Study Group and PECARN ^1^Department of Pediatrics, Division of Pediatric Emergency Medicine, Emory University School of Medicine, Atlanta, Georgia ^2^Children's Healthcare of Atlanta, Atlanta, Georgia ^3^Department of Pediatrics, Nemours Children's Health Delaware and the Sidney Kimmel Medical College at Thomas Jefferson University, Wilmington, Delaware ^4^Nationwide Children’s Hospital, Columbus, Ohio ^5^Children’s National Hospital, The George Washington University School of Medicine and Health Sciences, Washington DC ^6^Texas Children’s Hospital and Baylor College of Medicine, Houston, Texas ^7^Children’s Hospital Los Angeles and Keck School of Medicine of the University of Southern California, Los Angeles, California ^8^Children’s Hospital of Philadelphia, Philadelphia, Pennsylvania ^9^Washington University in St. Louis, St. Louis, Missouri ^10^Division of Pediatric Hematology/Oncology/BMT, Department of Pediatrics, Emory University School of Medicine, Atlanta, Georgia ^11^Aflac Cancer and Blood Disorders Center, Children’s Healthcare of Atlanta, Atlanta, Georgia ^12^Medical College of Wisconsin and Children’s Wisconsin, Milwaukee, Wisconsin ^13^University of California, San Francisco, California ^14^University of Utah School of Medicine, Salt Lake City, Utah ^15^Center for Maternal–Fetal Precision Medicine, University of California, San Francisco, California ^16^Department of Pediatrics and UCSF-Benioff Children's Hospital-Oakland, Oakland, California
Name and contact information for the trial sponsor {5b}	Claudia R. Morris, MD Professor of Pediatrics and Emergency Medicine Emory University School of Medicine 1760 Haygood Drive NE, W458 Atlanta, GA 30322, USA Email: claudia.r.morris@emory.edu
Role of sponsor {5c}	The funders had no role in the design and conduct of the study, the collection, management, analysis, and interpretation of the data, or the preparation, review, approval of the manuscript, or decision to submit the manuscript for publication

### Introduction

#### Background and rationale {6a}

Vaso-occlusive pain episodes (VOE) due to the sickling of erythrocytes are a clinical hallmark of sickle cell disease (SCD) [1]. SCD-VOEs are a common and debilitating medical emergency for children with SCD. VOE is a leading cause of emergency department (ED) visits and hospital admissions among children with SCD [2, 3]. There are approximately 70,000 ED visits for SCD-VOE each year in the USA among children and approximately 70% of these visits result in hospital admissions [4, 5]. Additionally, up to 20% of children with SCD experience three or more ED visits each year [3].

Despite the substantial illness burden and healthcare utilization conferred by SCD-VOE, disease-modifying therapies to treat SCD-VOE are lacking. Thus, clinicians rely on non-specific supportive therapies such as opioid analgesics and hydration for pain relief for children with SCD-VOE. While opioid analgesia is presently necessary for the treatment SCD-VOE and has been endorsed in recommendations from the National Heart, Lung, and Blood Institute (NHLBI), the American Society of Hematology, and the American College of Emergency Physicians [6, 7], it is associated with significant side effects. In addition, optimal efficacy is difficult to obtain because adherence to the timeliness and dosing of recommended therapies has been suboptimal in clinical practice [8, 9].

Several clinical trials of inhaled nitric oxide, intravenous magnesium, and agents that improve microvascular blood flow have failed to demonstrate effective pain reduction for SCD-VOE [10–12]. However, a single-center, double-blind, randomized, placebo-controlled trial that assessed the efficacy of the intravenous administration of the amino acid arginine demonstrated a 54% reduction in the use of parenteral opioids and potential reduction in hospital admission duration during SCD-VOE among 56 children in the USA [13]. Additionally, a double-blind randomized controlled trial of oral arginine demonstrated a reduction in the need for analgesic medications, shorter time to pain resolution with a nearly 48 h decrease in length of hospital stay, decreased blood pressure, and improved cardiopulmonary function among 68 children at two sites in Nigeria [14–16]. Moreover, the administration of arginine improves mitochondrial function and diminishes oxidative stress in children with SCD-VOE [17], in addition to increasing production of the vasodilator nitric oxide [18], which provides some insight into the potential mechanisms through which arginine may alleviate pain during SCD-VOE.

### Objectives {7}

Given the urgent need to develop disease-modifying therapies for SCD-VOE and the promising results from smaller trials of arginine therapy, the Sickle Cell Disease Treatment with Arginine Therapy (STArT) Trial aims to provide definitive evidence regarding the potential efficacy of intravenous arginine to treat SCD-VOE among children. The primary aim of the STArT trial is to determine the efficacy of intravenous arginine therapy to reduce time to crisis resolution, in children with SCD-VOE compared to placebo. Secondary aims of the STArT Trial include assessing the impact of arginine therapy on total parenteral opioid use, pain score reduction, patient-reported outcomes, and impact on adverse effects. Additionally, this trial aims to characterize alterations in the arginine metabolome and mitochondrial function in children with SCD-VOE. We hypothesize that intravenous arginine will reduce time to SCD-VOE crisis resolution, reduce total parenteral opioid use among children with SCD, and improve mitochondrial function.

### Trial design {8}

**Fig. 1:**
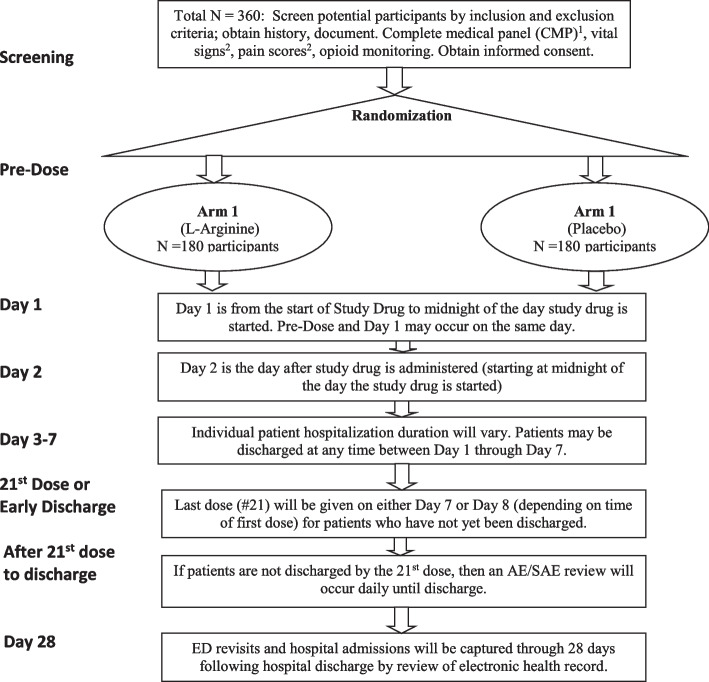
Schematic overview of the Sickle Cell Disease Treatment with Arginine Therapy (STArT) Trial 1 - A CMP will be obtained Pre-Dose. Last CMP in medical record will be reviewed prior to randomization (up to 12 months): if normal, the patient can be randomized prior to CMP results available. If past CMP is abnormal (meeting criteria for discontinuation of study drug) or not available, the results of the Pre-Dose CMP must be reviewed before randomization. This strategy avoids delay of first dose of study drug that arises from awaiting on the laboratory CMP report, while addressing safety. Patients with a CMP that meet criteria for discontinuation of study drug will be withdrawn from participation or have study drug withdrawn if already randomized. Patients with ALT > 3X upper limit, creatinine >1.0 or bicarb of 16 pre-dose will have CMP repeated in 24 hours 2 - The first set of Vital Signs (including oxygen saturation) and Pain scores will be captured when the patient first presents to the Emergency Department

STArT is a double-blind, placebo-controlled, randomized, phase 3, multicenter trial of intravenous arginine therapy in children with SCD-VOE. Figure 1 contains an overview of the trial design. The study is being conducted under an active Investigational New Drug (IND) #66943 (Sponsor –Morris, CR) and was registered in ClinicalTrials.gov (NCT04839354). This protocol manuscript was written in accordance with the Standard Protocol Items: Recommendations for Interventional Trials (SPIRIT) guidelines (Table 1 and Additional File 1) [19] and the Consolidated Standards of Reporting Trials (CONSORT) statement [20].

**Table 1:** Schedule of enrollment, interventions, and assessments

	**Screening**	**Pre-dose**^**1**^	**Day 1**^**2**^	**Day 2**^**3**^	**Days 3–7**^**4**^	**21st dose or early discharge**^**5**^	**After 21st dose through extended discharge (if applicable)**^**6**^	**Discharge**	**7–12 days post-discharge**	**Day 28**^**7**^
Inclusion and exclusion	✕	✕								
Demographics and genotype		✕								
Study treatment assignment		✕								
Medical history	✕	✕								
Pain crisis start date and time^a^		✕								
Time of last IV opioid/pain crisis end date and time^b^						✕	✕	✕		
Number of ED visits/hospitalizations in last year (excluding current)		✕								
ED admission information		✕								
Time of IV placement		✕								
Conmeds		✕	✕	✕	✕	✕	✕	✕		
Research labs^c^		✕		✕		✕		✕		
CMP^d^		✕	_✕_e	_✕_e	_✕_e					
Beta HCG^f^		✕								
Vital signs^g^	✕		✕	✕	✕	✕		✕		
Pain Score^g^	✕		✕	✕	✕	✕		✕		
PROs (PROMIS/Peds) QL/ASCQ-Me (adults)		_✕_h				✕			✕	
Symptoms Questionnaire^i^		✕	✕	✕	✕	✕	_✕_m	✕		
Opioid monitoring^j^	✕	✕	✕	✕	✕	✕	✕			
Maintenance fluid monitoring^j^		✕	✕	✕	✕	✕	✕			
AE^k^		✕	✕	✕	✕	✕	_✕_m			✕
SAE^k^		✕	✕	✕	✕	✕	✕			✕
Study drug administration^l^			✕	✕	✕	✕				

^1^Pre-dose is all information that is collected before the start of study drug

^2^Day 1 is from the start of study drug to midnight of the day study drug is started. Pre-dose and day 1 may occur on the same day

^3^Day 2 is the day after study drug is administered (starting at midnight of the day the study drug is started)

^4^Individual patient hospitalization duration will vary. Patients may be discharged at any time between day 1 and day 7

^5^21st dose may occur on either day 7 or day 8 (depending on time of first dose) for those who have not yet been discharged prior to 21st dose

^6^Assessments will only be done if hospital stay is extended past the 21st dose

^7^ED revisits and hospital admissions will be captured through 28 days following hospital discharge

^a^Pain crisis start date/time is defined as the date/time the patient started experiencing pain (this could be before ED visit)

^b^Pain crisis end date/time is defined as the date/time of last dose of parenteral opioid delivery

^c^Research labs: will be obtained with routine daily blood draws whenever possible and may be drawn on days when the research and clinical team suspect the patient may be discharged to avoid multiple venipunctures. In the event the patient is not discharged on that date, discharge labs should be drawn again prior to discharge. Up to eight research labs may be drawn in case of a prolonged hospital stay. Baseline labs to be drawn before the start of study drug

^d^All participants will receive a screening/baseline CMP prior to randomization. Participants that show normal CMP in the medical record within the last 12 months can be randomized before receiving the CMP results. If participants show abnormal CMP (any values with higher than protocol defined cutoffs for study drug discontinuation) in the medical record within the last 12 months, the results MUST be reviewed PRIOR to randomization. Patients with pre-dose CMP that meet criteria for discontinuation of study drug will be withdrawn from participation or have study drug discontinued if already randomized

^e^Randomized patients with ALT > 3 × upper limit, creatinine > 1.0 or bicarb of 16 pre-dose will have CMP repeated in 24 h and every other day during study drug administration until values are ALT < 3 × upper limit, creatinine < 1.0 or bicarb of > 16

^f^Pregnancy test obtained at pre-dose for females of reproductive age that have attained menarche

^g^The first set of Vital Signs (including oxygen saturation) and Pain scores will be captured when the patient first presents to the emergency department. The highest and lowest pain scores will be recorded from day 1 until the day the patient receives their 21st dose (or within 6 h of discharge). The first set of vital signs after 6 am will be collected up to the 21st dose/discharge, whichever comes first. Ensure oxygen saturation is captured at pre-dose and within 6 h of discharge

^h^Pre-dose PRO must be collected within ∼12 h

^i^Symptoms Questionnaire will be completed daily through day 9

^j^Use of parenteral opioids will be documented every shift by nursing staff per standard protocol, including opioids delivered by the PCA device as well as any intravenous, intranasal, and/or oral opioids used. Data on daily opioid use will be collected and recorded

^k^AEs will be monitored daily through day 9 or hospital or ED discharge (whichever comes first) and SAEs will be monitored daily through discharge; Return ED visits and hospitalizations will be followed for 28 days after hospital discharge

^l^Patients will be randomized within 12 h of their initial dose of parenteral opioid in the ED or ward and arginine will be administered TID

^m^Completed through day 9 only

## Methods: participants, interventions, and outcomes

### Study setting {9}

This trial is being conducted at 10 sites in the Pediatric Emergency Care Applied Research Network (PECARN), which is an established network of academic EDs in the USA who treat children. The sites were selected because they are (1) proven partners in PECARN and (2) provide clinical care for at least 100 children who present with SCD-VOE each year. Site selection was conducted through a screening process funded by the NHLBI (R34HL122557) in which 20 sites provided the annual number of clinical encounters among children who presented with SCD-VOE [5, 8]. The 10 selected sites include Children’s Healthcare of Atlanta at Egleston (Atlanta, GA), Children’s Healthcare of Atlanta at Hughes Spalding (Atlanta, GA), Children’s Hospital of Los Angeles (Los Angeles, CA), Children’s Hospital of Philadelphia (Philadelphia, PA), Children’s National Hospital (Washington D.C.), Children’s Hospital of Wisconsin (Milwaukee, WI), Nationwide Children’s Hospital (Columbus, OH), Texas Children’s Hospital (Houston, TX), UCSF Benioff Children’s Hospital (San Francisco, CA), and St. Louis Children’s Hospital (St. Louis, MO).

### Eligibility criteria {10}

Participants include all children, adolescents, and young adults with known SCD of any genotype, aged 3 to 21 years who require medical care in an acute care setting (e.g., ED, hospital ward, day hospital, or clinic) for pain attributable to an SCD-VOE and require parenteral opioid analgesic therapy. SCD-VOE is defined as pain not attributable to non-SCD-related issues (e.g., forearm fracture, head trauma) in a child with known SCD.

Participants with SCD-VOE are excluded from the trial if they respond to two or fewer doses of intravenous opioids sufficiently to preclude the need for hospital admission, if > 12 h passed between the first dose of intravenous opioids to treat SCD-VOE and consent, if their hemoglobin is < 5 g/dL or they need emergent packed red blood cell transfusion due to hemodynamic instability, including hypotension or septic shock, if they have acute mental status or neurologic changes, if they have an acute stroke or concern for stroke, if they received ketamine in the ED as a therapy for SCD-VOE, or if they have received glutamine within 30 days. Additionally, participants with SCD-VOE are excluded if they have started taking a new drug for SCD within 3 months (e.g., hydroxyurea, voxelotor, crizanlizumab), if they have had ≥ 3 ED visits in which they received parenteral opioids within 7 days (excluding current visit), if they were discharged from the hospital within 7 days, if they were previously enrolled in this trial, or if they have used inhaled nitric oxide, sildenafil, or arginine within 30 days of enrollment. Patients with SCD-VOE are also excluded if they do not speak English or Spanish, are pregnant, are allergic to arginine, or are aged > 18 and lack medical decision-making capacity to provide informed consent.

### Who will take informed consent? {26a}

A waiver of authorization was put in place to facilitate the process of pre-screening to determine eligibility based on the medical record prior to approaching potential participants both for current and future encounters in the ED. Written permission from parents or legal guardians is required for participation for patients who are eligible for this trial and are aged < 18 years. Emancipated minors are not enrolled without parental consent. Patients aged ≥ 18 years provide written consent for themselves. Parents, guardians, or participants aged ≥ 18 years are informed about the objectives of the study as well as potential risks and benefits of participation. Documentation of consent is kept in either paper or electronic forms and is maintained in encrypted, password-protected files by trial staff.

Children who can provide assent and who are competent for decision-making are asked to give assent to the study procedures after an age-appropriate discussion of risks and benefits. Child assent is waived if the child is aged ≤ 6 years, has mentation that is not developmentally age-appropriate, has decreased level of consciousness, or has psychiatric problems.

### Additional consent provisions for collection and use of participant data and biological specimens {26b}

Additional consent is obtained for inclusion in the SCD-VOE biorepository.

## Interventions

### Explanation for the choice of comparators {6b}

Both arms of the trial will receive standard care for SCD-VOE, which includes parenteral opioids, non-steroidal anti-inflammatory drugs, and IV fluids as needed based on the 2014 NHLBI guidelines for the treatment of acute vaso-occlusive pain in SCD [6]. Arginine is a safe nutritional supplement approved by the U.S. Food and Drug Administration in parenteral form for growth hormone stimulation testing and has nearly 50 years of safety experience through its use in clinical practice. Intravenous saline will be administered as the comparator, which is safe, has no side effects, and has no substantial therapeutic benefit.

### Intervention description {11a}

Participants are randomized to one of two study arms and receive either (1) a one-time loading dose of L-arginine (200 mg/kg with a maximum of 20 g) administered intravenously in the ED or inpatient unit followed by a standard dose of 100 mg/kg (maximum 10 g) three times a day or (2) a one-time placebo (normal saline) loading dose of 2 ml/kg administered intravenously followed by 1 ml/kg three times a day for 21 doses or until discharge, whichever comes first. The administered volume of fluids is equivalent in these two arms.

Prior studies have demonstrated that arginine levels are low in both adults and children with SCD-VOE [21, 22]. Prior studies have also demonstrated a dose-dependent response of arginine on nitric oxide metabolite production [18, 23] and improved mitochondrial function [17] in children hospitalized with SCD-VOE. Lower doses of arginine are likely to be subtherapeutic in SCD-VOE as reported in other conditions of endothelial dysfunction [24]. Moreover, higher levels of plasma arginine are likely needed to overcome the impact of arginase (the enzyme that degrades arginine), to overcome greater arginine consumption or metabolism during SCD-VOE, and to achieve adequate global arginine bioavailability ratios [25, 26]. Intravenous doses of arginine as high as 500 mg/kg (maximum 30 g) are safe and are commonly used for growth hormone stimulation testing [27, 28] and hyperammonemia [29].

Prior to enrollment, a complete metabolic panel is obtained to identify participants who may have significant liver or renal dysfunction, and the medical record is reviewed to identify participants who may be at risk for the same, prior to the availability of these results. Additional laboratory monitoring occurs during the trial (Table 2).

**Table 2:** Laboratory monitoring in the STArT Trial

	Pre-dose ^1^	Day 1 ^2^	Day 2 ^3^	Days 3–7	21st dose or discharge
Complete blood count (CBC) with reticulocyte count (clinical)*	X		X		X
Comprehensive metabolic panel (CMP)	X**	X***		X***	
BHCG (in females aged ≥ 13 years)	X				
Research labs	X		X		X

^1^Pre-dose is before the start of study drug. Pre-dose and day 1 may occur on the same day

^2^Day 1 is from the start of study drug to midnight of the day study drug is started

^3^Day 2 is the day after study drug is administered (starting at midnight of the day the study drug is started)

^*^When available clinically

^**^A CMP will be obtained pre-dose. Last CMP in medical record will be reviewed prior to randomization (up to 12 months): if normal, the patient can be randomized prior to CMP results available. If past CMP is abnormal (meeting criteria for discontinuation of study drug) or not available, the results of the pre-dose CMP must be reviewed before randomization. Patients with CMP that meet criteria for discontinuation of study drug will be withdrawn from participation or have study drug discontinued if already randomized

^***^Randomized participants with ALT > 3 times the upper limit of normal, creatinine > 1.2 mg/dL, or acidosis defined as a bicarbonate level of < 16 mEq/L before the first dose will have a CMP repeated in 24 h and every other day during the study drug administration until values are ALT < 3 times the upper limit of normal, creatinine < 1.2 mg/dL or bicarbonate level > 16 mEq/L

### Criteria for discontinuing or modifying allocated interventions {11b}

The study drug will be discontinued if a participant meets any of the following criteria: neurologic dysfunction or development of stroke, allergic reaction to the study drug, participant or caregiver request, or abnormal lab results including an alanine transaminase (ALT) level > 6 times the upper limit of normal, a creatinine level of > 1.2 mg/dL, or acidosis (i.e., bicarbonate < 16 mEq/L). In these cases, the study drug is stopped but clinical data and research blood collection will continue.

### Strategies to improve adherence to interventions {11c}

IV arginine will be administered while participants are admitted to the hospital for SCD-VOE. Thus, we anticipate that adherence will be high as participants will still receive parenteral opioids as needed for pain related to SCD-VOE.

### Relevant concomitant care permitted or prohibited during the trial {11d}

Participants are allowed to request parenteral opioids for SCD-VOE per the standard of clinical care. IV fluids will be administered in accordance with NHLBI and hospital guidelines. No non-experimental therapies will be prohibited during the trial.

### Provisions for post-trial care {30}

There is no anticipated harm and compensation for trial participation. All post-trial care will be provided according to current practice at each institution. As IV arginine will be administered during the hospital admission, we do not anticipate post-trial care requirements beyond the standard of care.

### Outcomes {12}

The STArT Trial’s primary outcome is time to SCD-VOE crisis resolution. This is defined as the time (in hours) from first study drug delivery to the last dose of parenteral opioid delivery. Length of hospital stay, measured from the time of presentation to the ED to the time of hospital discharge, will also be analyzed for additional insight in relation to the primary outcome.

Secondary outcomes include total parenteral opioid use, change in pain score, and changes in certain patient-reported outcomes (PROs). The outcome of total parenteral opioid use is measured in intravenous morphine equivalents (mg/kg) and is measured from the time of first study drug delivery to the final opioid administered. Change in numerical pain intensity score from presentation to discharge is measured through documentation of the highest and lowest daily pain scores recorded on a scale from 0 to 10 by clinical nursing staff. Three PROs are measured within 12 h of first study drug delivery and again on the day of hospital discharge [30–34]. These PROs include the Patient-Reported Outcome Measurement Information System (PROMIS) Pain Interference score (i.e., used to measure the extent pain interferes with activities), the PROMIS Pain Behavior (i.e., used to measure behaviors that indicate an individual is experiencing pain), and the PROMIS Fatigue (i.e., used to assess the impact and experience of fatigue during the week preceding its administration). These PROs are also assessed 7–12 days after hospital discharge. Tertiary/exploratory outcomes include a question about general pain improvement, chronic pain, oxygen saturation at discharge, medication quantification score, and other PROs.

Blood samples are collected for amino acid analysis, arginase activity/concentration, nitric oxide metabolites, mitochondrial function, and biomarkers of oxidative stress at the time of other clinical lab collections whenever possible to minimize patient discomfort, prior to study drug delivery, day 2 and on the day of discharge (or last day of study drug delivery and then day of discharge for patients with a prolonged hospital stay). Additional blood is collected to answer future mechanistic questions about SCD-VOE.

### Participant timeline {13}

The schematic overview of the STArT Trial is shown in Fig. 1. Study start-up began on September 1, 2020, including protocol development, single institutional review board approval, data safety monitoring board (DSMB) review and approval, and local site IRB approval. Trial enrollment began on June 21, 2021, and it is anticipated that enrollment will continue for approximately 5 years from that date. Participants are enrolled in an acute care setting when they present for an SCD-VOE where initial pain management begins, or within 12 h of receiving their first dose of intravenous opioids. Focused recruitment in the ED is crucial for successful enrollment because guardians may not be available once a patient is transferred to the inpatient unit.

### Sample size {14}

Based on the results of a previous, single-center trial of arginine [13], the difference in time to crisis resolution between the intervention arm and the placebo arm is anticipated to be 17 h. A Type I error bound of 0.05 (alpha) will be used and age is assumed to not be informative of the outcome (i.e., a conservative bound for sample size). A simulation-based approach was used to determine the sample size required to detect a shift in distribution of 17 h with 85% power. To simulate the outcome of placebo administration, time-to-crisis resolution data from a previous trial of magnesium for SCD-VOE was used [11]. For the intervention arm, outcomes were simulated from the same distribution, shifted down by 17 h, and 5000 trials were simulated. This process resulted in a required sample size of 166 participants per arm. Accounting for an anticipated ~ 1% withdrawal rate based on experience with prior IV arginine studies [13, 35], interim efficacy monitoring (with inflation of 2%), and crossover/non-adherence of 3% (e.g., participants randomized to receive arginine did not receive the indicated amount) resulted in an overall inflation of 8%, or total of 180 participants per arm.

### Recruitment {15}

ED-based recruitment staff available to obtain consent prior to admission is essential for successful enrollment into acute VOE studies since guardians are often not available once the child is transferred to the inpatient unit. Recruitment and study procedures will occur during “down” times when the patient is not actively engaged in treatment (e.g., waiting for testing to commence and/or results to return) to avoid interfering with clinical care.

## Alignment of interventions: allocation

### Sequence generation {16a}

Participants are randomized 1:1 to receive either intravenous arginine or placebo (i.e., intravenous normal saline). Permuted blocks, stratified by site and age group (i.e., 3–11 years and 12–21 years), were created by the Emergency Medical Services for Children Data Center (EDC) housed at the University of Utah prior to trial start. The system utilizes the participant’s age group to deliver the next assigned treatment through a randomization number.

### Concealment mechanism {16b}

Online randomization tables are available to each site’s pharmacy to facilitate treatment allocation. These tables are not available to research staff, nurses, or clinicians to maintain blinding.

### Implementation {16c}

The allocation sequence was generated by the EDC. ED-based recruitment staff enroll participants. Participants are randomly assigned to interventions according to random numbers provided to pharmacists at each site.

## Assignment of interventions: blinding

### Who will be blinded {17a}

The pharmacist who distributes the study medication or placebo is unblinded to the treatment, while treating clinicians, nurses, and participants remain blinded. The intravenous arginine that is administered is prepared and appropriately blinded (i.e., study drug is labeled as investigational and contains appropriate participant identifiers) from clinical staff at each site. Data analysts will be blinded during the trial for interim analyses until trial completion. At the completion of the trial, the data will be unblinded. All sites are responsible for documentation of drug storage and dispensing information.

### Procedure for unblinding if needed {17b}

In the event of adverse events thought to be related to arginine providers will assume that the subject is receiving active drug (arginine) and take appropriate steps. However, the clinical team and investigators will not attempt to unblind themselves.

## Data collection and management

### Plans for assessment and collection of outcomes {18a}

The STArT Trial uses standardized data collection forms through REDCap for information on screening and enrollment, survey responses, and safety measures. The web-based interface is designed specifically for clinical trials and prospective observational studies. REDCap and the electronic health record will be used for all data collection.

### Plans to promote participant retention and complete follow-up {18b}

The trial drug will only be administered during hospital admission so it is anticipated that challenges to participant retention will be minimal. ED revisits and hospital readmissions through day 28 after index hospital discharge will be assessed through review of the electronic health record at each site.

### Data management {19}

Data management is done through the EDC housed at the University of Utah. The EDC has developed a Query Management System that allows for data checks on individual data fields (e.g., data that are missing or out of range for continuous variables) and conducts data validation within and between the forms used in the STArT Trial.

### Confidentiality {27}

All REDCap data collection forms are password-protected, and data are coded with a number to ensure participant confidentiality.

### Plans for collection, laboratory evaluation and storage of biological specimens for genetic or molecular analysis in this trial/future use {33}

Understanding a drug mechanism-of-action is imperative together with assessing clinical outcomes. In this trial, additional blood is collected to answer mechanistic questions related to arginine metabolism and future mechanistic questions about SCD-VOE, creating what will be the largest biorepository of blood samples from patients hospitalized with SCD-VOE. Blood (for plasma, erythrocytes, and platelets) will be drawn, processed, and stored for future batched analyses at the time of presentation prior to study drug delivery (pre-dose), on day 1, and prior to discharge for future ancillary studies. Additional consent is obtained from participants prior to the collection of any blood samples stored in the biorepository for future mechanistic studies.

## Statistical methods

### Statistical methods for primary and secondary outcomes {20a}

The primary outcome of time-to-crisis resolution for SCD-VOE will be analyzed with the Van Elteren test, stratified by site and age group, using an overall significance level of *P* < 0.05 (adjusted based on interim analyses). Additionally, the distribution of the primary outcome in each trial arm will be measured with mean, median and interquartile range and the difference in medians will be reported with a 95% confidence interval. The secondary outcomes of total parenteral opioid use (in mg/kg), change in numerical pain intensity scores, and PRO scores will be analyzed through the same approach as the primary outcome. However, PRO measures will not be stratified by randomization age groups but rather the age groups used to determine the survey type. Holm’s method will be used to adjust for multiple comparisons of secondary outcomes. Analyses of safety outcomes including acute chest syndrome, requirement for blood transfusion, supplemental oxygen requirement, 28-day return visits to the ED, and clinical worsening will be conducted using the Mantel–Haenszel chi-square test, stratified by clinical center and age group. All analyses will be undertaken by the intention-to-treat principle and all analyses will be two-sided.

### Interim analyses {21b}

The DSMB will meet for two formal interim efficacy analyses of the primary and secondary outcome measures after 150 and 250 subjects have been enrolled. The DSMB will also review safety information at least annually. The DSMB can recommend whether to terminate enrollment in STArT because of potential safety concerns or feasibility issues. The DSMB will also examine results from interim data analyses to decide whether to terminate the trial based on high evidence of efficacy or low likelihood of benefit.

### Methods for additional analyses (e.g., subgroup analyses) {20b}

The formal subgroups specified for this trial are age group, use of hydroxyurea, and participants’ sex. The interaction between assigned treatment and each subgroup factor in a linear model will be tested (significance level of *P* < 0.017). The model will include a main effect for treatment, subgroup factor, and an interaction between subgroup and treatment. Secondary outcomes will also be assessed in similar models.

### Methods in analysis to handle protocol non-adherence and any statistical methods to handle missing data {20c}

High levels of missing data for the outcomes of interest are not expected because these are readily available, frequently documented in medical records, and are short term. Should there be high rates of participant attrition in the STArT Trial, baseline characteristics and available information on the hospital course will be reviewed and compared to participants who do not withdraw from participation to assess for any differences in those who withdraw and those who do not.

### Plans to give access to the full protocol, participant-level data and statistical code {31c}

A dataset will be produced with participant-level, de-identified data in accordance with the Health Insurance Portability and Accountability Act (HIPAA) and will be made available to the public. Statistical code used for these analyses will be made available through the EDC for reasonable requests.

## Oversight and monitoring

### Composition of the coordinating center and trial steering committee {5d}

The trial steering committee is comprised of senior investigators and statisticians with expertise in clinical trials, pediatric emergency medicine research, and SCD. The trial steering committee’s responsibilities include assessment of enrollment progress and coordination among the sites. The EDC is housed at the University of Utah’s Data Coordinating Center and has been federally funded since 2001 by the Health Resources and Services Administration’s (HRSA) Emergency Medical Services for Children (EMSC) program. The EDC oversees data collection, quality assurance, and data quality control for each of the seven PECARN nodes and their hospital affiliates. Funding provides PECARN nodes with access to EDC faculty and staff consisting of faculty and master statisticians, clinical project managers, clinical data managers, program directors, information technology and business intelligence tools and software. There is a trial retention committee comprised of senior investigators and EDC members that meet twice a year as needed. The trial retention committee’s responsibility is to address recruitment challenges faced during the trial. Site principal investigators are responsible for all aspects of local organization including identifying potential participants and overseeing consent and enrollment of participants.

### Composition of the data monitoring committee, its role and reporting structure {21a}

The STArT Trial utilizes an independent NHLBI-appointed Sickle Cell Disease DSMB. The purpose of the DSMB is to advise the funding agency (i.e., NHLBI) and the STArT Trial principal investigator regarding the continuing safety of study participants and the continuing validity and scientific merit of the study. The DSMB is responsible for monitoring accrual of study subjects, adherence to the protocol, assessment of data quality, performance of individual clinical sites, review of serious adverse events, and other participant safety issues.

### Adverse event reporting and harms {22}

A daily symptoms questionnaire is collected while participants receive study drug and on the day of discharge (Supplemental Fig. 1). Frequency and intensity of symptoms will be assessed for all adverse events by querying the medical record, the clinical team, the patient, and/or their parent/guardian. All adverse events that occur after randomization through hospital discharge are included. Any adverse event will be assessed for relatedness to the study by a site principal investigator or designated co-investigator coded according to the latest version of the Medical Dictionary for Regulatory Activities [36].

### Frequency and plans for auditing trial conduct {23}

There is a monthly call among all site principal investigators at each site, co-investigators, site research coordinators, and the EDC staff including the program manager, biostatisticians, and other support staff. Additionally, there is a bi-monthly call for the EDC, the NHLBI program officer assigned to this UG3/UH3 grant that funds the clinical trial, and the study principal investigator. This trial is also discussed at quarterly PECARN steering committee meetings. Locally, site investigators (including pediatric emergency medicine investigators and pediatric hematology investigators) meet with all local research staff to discuss this trial among other ongoing PECARN studies.

### Plans for communicating important protocol amendments to relevant parties (e.g., trial participants, ethical committees) {25}

Frequent communication between key stakeholders, the site investigators, and key research staff at participating sites occurs throughout the study. As necessary, the EDC will contact key research staff when preparing submissions to the University of Utah IRB, which is the IRB of record. This includes main protocol amendments, individual site amendments, and continuing reviews. Participating sites communicate with their local IRBs and upload approvals to eRoom or Florence eBinder.

The EDC will prepare meeting minutes and share them with the lead and participating sites. Topics for these meetings will be generated from an ongoing feedback loop of communication regarding all aspects of the study. This constant interaction will allow identification of problems early in the study so that solutions can be implemented, compliance can be enhanced, safety can be maintained, and any other problems can be corrected.

### Dissemination plans {31a}

The results of the STArT Trial will be disseminated to the public through peer-reviewed publication and through timely posting of trial results in ClinicalTrials.gov, in accordance to the National Institutes of Health requirement to post results to ClinicalTrials.gov within 12 months of trial completion [37]. Publication will be sought through open access publication to facilitate broad access to the STArT Trial’s findings. Authorship in all resulting publications will be assigned according to the International Committee of Medical Journal Editors guidelines.

## Discussion

The STArT Trial will provide definitive evidence for whether intravenous arginine is effective for the treatment of SCD-VOE among children, adolescents, and young adults aged 3–21 years. Currently, the treatment of SCD-VOE relies on supportive care including opioid pain medications, non-steroidal anti-inflammatory drugs, and intravenous fluids with variable strength of evidence [6, 38, 39]. Arginine therapy has demonstrated opioid and analgesic-sparing benefits in phase 2 trials [13, 14]. Should intravenous arginine prove effective at reducing pain during SCD-VOE, opioid medications use may be reduced, sparing potential side effects including constipation, hyperalgesia, drug tolerance, adverse impact on brain function, and the risk for respiratory depression may be minimized. Additionally, the risks of opioid dependence and tolerance may also be modified, although the expected magnitude of the effect may limit this benefit to select patients.

Emerging data supports long-term multi-organ side effects of opioids in SCD beyond the acute symptoms not appreciated years ago, and the recurrent/chronic use of large doses of opioids compounds the problem further [40]. Opioid-induced endothelial-, mast cell-, renal mesangial-, and epithelial-cell-specific effects and proinflammatory signaling has been reported [40]. Experts believe that opioids have organ-specific pathological effects. Experimental and clinical studies, even though extremely few, suggest that opioids may exacerbate existing organ damage and stimulate pathologies of their own [40]. Opioid-sparing pain therapies are needed; decreasing opioid use is a meaningful effect on a clinically important outcome of SCD-related morbidity that may be provided through arginine replacement therapy.

**Fig. 2:**
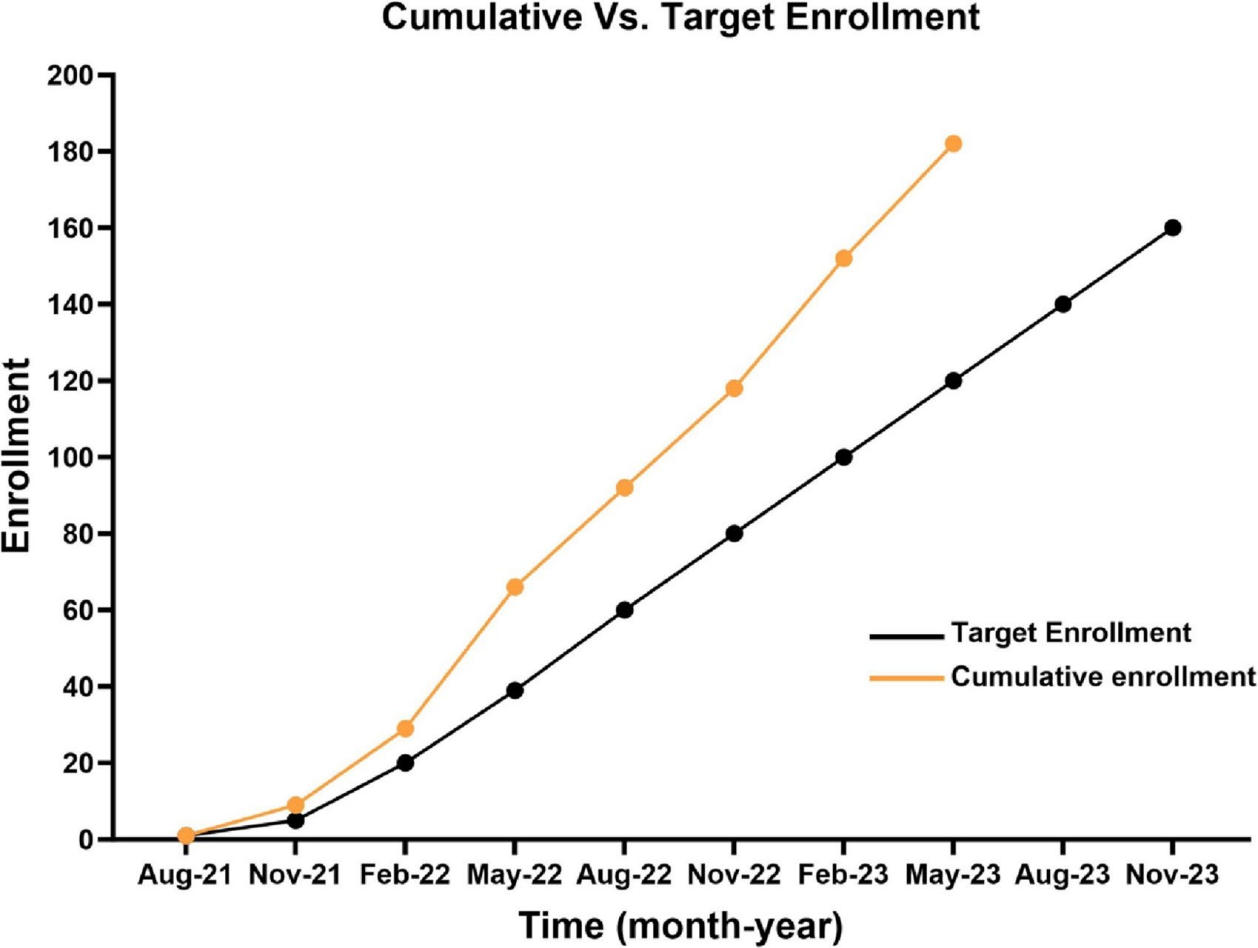
Enrollment in the STArT Trial from June 21, 2021, to May 31, 2023, with a goal of 360 subjects enrolled before April 2026. Cumulative enrollment has consistently surpassed target enrollment milestones

Previous trials to treat pain from SCD-VOE have encountered multiple challenges that we accounted for in the design of the STArT Trial. First, several multicenter trials in rare diseases, including SCD, have terminated prematurely due to lack of sufficient patient accrual [40–42]. Enrollment of participants into trials that are terminated prematurely results in exposure to therapies without resulting knowledge of potential benefit or harm from the studied therapy. Building on the strength of established partnerships between committed investigators in Emergency Medicine and Hematology developed through a previous trial in PECARN [11], we will conduct this trial at 10 sites in PECARN. A previous trial for SCD-VOE in PECARN was one of the few trials for SCD-VOE to accrue patients in the goal enrollment period [11, 43–45]. Additionally, pre-screening and pre-enrollment consenting in Hematology clinics use substantial resources and only a proportion of children, adolescents, and young adults with SCD who are seen in clinic develop SCD-VOE shortly thereafter. Enrollment of children, adolescents, and young adults with SCD-VOE in the ED surpasses this challenge and was successfully implemented in three prior trials [11, 13, 36]. Enrollment in the ED through research coordinators physically located in the ED allows for real-time education of patients and their caregivers and enables consent of caregivers while in the ED, as they often leave after hospital admission to attend to other issues in the home. Providing education about the study to all patients with SCD and their families while in the ED regardless of their eligibility offers families time to ask questions, review materials, and speak with their hematologists before consenting, a strategy that increases participation rates during a future ED visit, particularly since nearly 30% of ED treat-and-release visits for SCD pain episodes within PECARN sites have return ED visits within 14 days [45]. Pre-consent in the ED for future visits, and not just in hematology clinics, may also facilitate enrollment in future ED encounters. Building on these foundational approaches, we have surpassed enrollment goals as of publication of this protocol paper (Fig. 2).

Other challenges encountered in prior trials include the need for a clinically relevant, measurable, and patient-centered primary endpoint, which has varied in prior trials. Some have used total hospital length of stay [11, 12]. Consistent with prior studies [10, 46–48], we chose to use time to crisis resolution because this is a measurable patient-centered outcome that is agnostic to potentially unrelated factors that may influence hospital length of stay due to SCD-VOE (e.g., development of a fever, social issues such as self-care competency) [42]. Additionally, we opted to measure total parenteral opioid administered as a previous single-center study suggested an opioid-sparing effect of arginine for SCD-VOE [13].

The selection of a “minimum pain intensity reduction” has varied in previous trials. For example, a reduction in pain intensity of 30–50% from the self-reported level prior to treatment has been used in some studies [48–50]. Other studies in adults have used visual analog scales with an upper limit of 100 mm [42, 50, 51]. As our study enrolled children, adolescents, and young adults aged 3-21, we opted to use age-appropriate measurements of numerical pain intensity scores. Moreover, in order to augment our understanding of the impact of arginine on SCD-VOE pain and the life of a participant and their family, we also include three PROs [30–33]. We hope that more comprehensive, and patient-centered outcomes will further understanding of how SCD-VOE impacts the lives of patients and how arginine can improve quality of life beyond a simple numerical assignment to describe one’s pain.

## Trial status

The STArT Trial (protocol v1.03 dated January 18, 2021) began recruitment on June 21, 2021, and enrollment will take place over 5 years with an estimated end date of February 28, 2026. To date, the STArT Trial has surpassed its enrollment goal. It is anticipated that this definitive trial will provide novel information about the efficacy of arginine for the treatment of SCD-VOE for children, adolescents, and young adults. The STArT Trial builds off established relationships between hematologists and emergency medicine providers to ensure adequate participant accrual to elucidate the efficacy of this novel therapy for the treatment of SCD-VOE pain.

### Supplementary Information

**Additional file 1: Supplemental Figure 1. **Symptom questionnaire administered daily to participants in the STArT Trial.* 

**Additional file 2. **Spirit checklist

## Data Availability

The trial statistician (TCC), lead data manager (MR), and study principal investigator (CRM) will have full, unrestricted access to and be responsible for the final dataset. After publication of the main and secondary results, a public use dataset will be made available through the National Heart, Lung, and Blood Institute. The study funders will not restrict access to study data.

## Abbreviations

ALT: Alanine transaminase
CONSORT: Consolidated Standards of Reporting Trials
DSMB: Data safety monitoring board
ED: Emergency department
HIPAA: Health Insurance Portability and Accountability Act
HRSA: Health Resources and Services Administration
IND: Investigational new drug
EDC: Emergency Medical Services for Children Data Center
EMSC: Emergency Medical Services for Children
NHLBI: National Heart, Lung, and Blood Institute
PECARN: Pediatric Emergency Care Applied Research Network
PROs: Patient-reported outcomes
PROMIS: Patient-Reported Outcome Measurement Information System
SCD: Sickle cell disease
STArT: Sickle Cell Disease Treatment with Arginine Therapy
SPIRIT: Standard Protocol Items: Recommendations for Interventional Trials
VOE: Vaso-occlusive episodes
