# Supplementary material for: Sickle Cell Disease Treatment with Arginine Therapy (STArT): study protocol for a phase 3 randomized controlled trial
Source: Trials. 2023 Aug 17;24:538. doi: 10.1186/s13063-023-07538-z (PMC10433602; doi:10.1186/s13063-023-07538-z)
Supplement: Supplementary file 1 — Additional file 1: Supplemental Figure 1. Symptom questionnaire administered daily to participants in the STArT Trial.* [file 13063_2023_7538_MOESM1_ESM.docx]

**Supplemental Figure 1.** Symptom questionnaire administered daily to participants in the STArT Trial.*

*Filled out by caregivers for participants aged 3 to 7 years and by participants aged 8 years and older.
